# Investigations on the Ability of the Insular Cortex to Process Peripheral Immunosuppression

**DOI:** 10.1007/s11481-024-10143-9

**Published:** 2024-07-30

**Authors:** Julia Bihorac, Yasmin Salem, Laura Lückemann, Manfred Schedlowski, Raphael Doenlen, Harald Engler, Melanie D. Mark, Kirsten Dombrowski, Katharina Spoida, Martin Hadamitzky

**Affiliations:** 1 Institute of Medical Psychology and Behavioral Immunobiology, Center for Translational Neuro- Behavioral Sciences (C-TNBS), University Hospital Essen, University of Duisburg-Essen, Essen, 45147 Germany; 2 Department of Clinical Neuroscience, Osher Center for Integrative Medicine, Karolinska Institutet, Stockholm, Sweden; 3 Center of Phenogenomics, School of Life Sciences, Ecole Polytechnique Fédérale de Lausanne, Lausanne, Switzerland; 4 Behavioral Neuroscience, Faculty for Biology and Biotechnology, Ruhr-University Bochum, Bochum, Germany; 5 Department of General Zoology and Neurobiology, Ruhr-University Bochum, Bochum, Germany

**Keywords:** Rapamycin, mTOR, Insular Cortex, DREADD, Interoception, CNO, CaMKIIa, cFos

## Abstract

The brain and immune system communicate through complex bidirectional pathways, but the specificity by which the brain perceives or even remembers alterations in immune homeostasis is still poorly understood. Recent data revealed that immune-related information under peripheral inflammatory conditions, termed as “immunengram”, were represented in specific neuronal ensembles in the insular cortex (IC). Chemogenetic reactivation of these neuronal ensembles was sufficient to retrieve the inflammatory stages, indicating that the brain can store and retrieve specific immune responses. Against this background, the current approach was designed to investigate the ability of the IC to process states of immunosuppression pharmacologically induced by the mechanistic target of rapamycin (mTOR) inhibitor rapamycin. We here show that the IC perceives the initial state of immunosuppression, reflected by increased deep-brain electroencephalography (EEG) activity during acute immunosuppressive drug treatment. Following an experienced period of immunosuppression, though, diminished splenic cytokine production as formerly induced by rapamycin could not be reinstated by nonspecific chemogenetic activation or inhibition of the IC. These findings suggest that the information of a past, or experienced status of pharmacologically induced immunosuppression is not represented in the IC. Together, the present work extends the view of immune-to-brain communication during the states of peripheral immunosuppression and foster the prominent role of the IC for interoception.

**Supplementary Information:**

The online version contains supplementary material available at 10.1007/s11481-024-10143-9.

## Introduction

For a long time, the central nervous system (CNS) and peripheral immune system were considered to be separate and autonomously acting entities (Tracey 2009). A growing body of evidence, however, has shown that the CNS not only has the capacity to affect peripheral immune functions but is also able to perceive and process signals from the immune system (Steinman 2004; Dantzer et al. 2008; Tracey 2010). Importantly, this neuroimmune communication is believed to underlie an immunoregulatory role of the brain and a sensory function of the immune system and occurs via both humoral and neuronal pathways (Dantzer 2018). Within the brain, especially the posterior part of the insular cortex (IC) is suggested to contain representations of the immune system. Since this brain region receives input from peripheral neurons that respond to immune signals (Goehler et al. 2000; Thayer and Sternberg 2009; Reardon et al. 2018) and due to the fact that it is associated with sympathetic afferent innervation and visceral information processing (Saito et al. 2012; Montalbano and Tubbs 2018; Méndez-Ruette et al. 2019), the IC is considered as primary cortical site of interoception (i.e. the sensing and integrating the body’s physiological state; (Craig 2002, 2003; Gogolla 2017).

Besides its role in perceiving changes in immunological homeostasis (Cohen et al. 1994), findings indicate that the IC is also the primary neuronal structure crucially involved in the acquisition and retrieval of taste-immune-associative learning and memory (Ramirez-Amaya et al. 1996; Ramirez-Amaya and Bermudez-Rattoni 1999; Pacheco-Lopez et al. 2005). By employing paradigms of classical or Pavlovian conditioning, the mere association of a novel sweet taste as conditioned stimulus (CS) with the injection of an immunosuppressive drug as unconditioned stimulus (US) has been shown to be sufficient to modulate peripheral immune responses upon re-exposure to the CS alone at a later time (Hadamitzky et al. 2020). The notion that the IC is largely involved in neuro-immune interactions is further supported by evidence demonstrating that pro-inflammatory conditions, for instance elicited by the administration of lipopolysaccharides, are accompanied by increased IC activity as well as altered functional connectivity (Goehler et al. 2000; Doenlen et al. 2011; Kerezoudis et al. 2022). A more recent approach revealed that the IC is able to store information specific to the anatomical location and nature of distinct peripheral inflammatory disease stage in the body. More important, these immunological ‘memory engrams’ which were formed during the phase of inflammation could restore the initial disease state when reactivated even after complete recovery (Koren et al. 2021). Based on these findings, and on the classical neuroscience concept of interoception, it has been suggested that the brain, most prominently the IC, receives inputs from the peripheral immune system to formulate a neuronal representation of the organism’s immunological state, a process recently defined as “immunoception” This concept implies that the brain continuously monitors alterations in immune activity and can, in turn, regulate the immune system to produce a physiologically coordinated response (Koren and Rolls 2022). This concept of “immunoception” is furthermore supported by approaches of taste-immune associative learning. Following association of a pharmacologically suppressed immune system with an external sensory cue (e.g., sweet saccharin solution), anew presentation of the cue alone at a later time point can elicit immunosuppressive responses such as suppressed T cell functioning and cytokine production (Ader and Cohen 1975; Hadamitzky et al. 2020). Importantly, a prominent modulatory role of the IC not only during the acquisition (learning) phase but also during the retrieval (memory) phase of a conditioned immunosuppression has been described in these paradigms as well (Ramirez-Amaya et al. 1996, 1998; Ramirez-Amaya and Bermudez-Rattoni 1999; Pacheco-Lopez et al. 2005). However, whether and to what extend a pharmacologically induced stage of immunosuppression is also centrally represented in the IC is unknown. Against this background, the current approach was designed to investigate the ability of the IC to process states of immunosuppression pharmacologically induced by the mechanistic target of rapamycin (mTOR) inhibitor rapamycin (Lückemann et al. 2019, 2021). This macrolide, also known as sirolimus, is a small-molecule immunosuppressive drug known to inhibit T and B cell proliferation, as well as splenic cytokine production (Sehgal 2003; Guertin and Sabatini 2009). Due to its immunogenicity rapamycin is efficient in preventing rejection of organ transplants (Vézina et al. 1975) and has been proven useful in reducing primary and metastatic tumor growth in experimental animals and humans (Guba et al. 2002; Guertin and Sabatini 2009; Lane and Breuleux 2009; Alain et al. 2010; Dancey 2010).

## Methods

### Animals

Male Dark Agouti rats (DA/HanRj, Janvier, France; 220–240 g, corresponding to 50–60 days of age) were kept under an inverse 12-hour light/dark cycle with lights off at 7 a.m. and lights on 7 p.m. to enable the experiments to be conducted during the activity phase of the rats’ awake/sleep cycle. After an acclimatization period of two weeks, experiments were started. The animal facilities and all experimental procedures were in accordance with National Institutes of Health and Association for the Assessment and Accreditation of Laboratory Animal Care guidelines and were approved by the Cantonal Veterinary Office of Zurich (Switzerland) and the Institutional Animal Care and Use Committee (LANUV Düsseldorf, North Rhine-Westphalia).

### Experimental Design

**Fig. 1:**
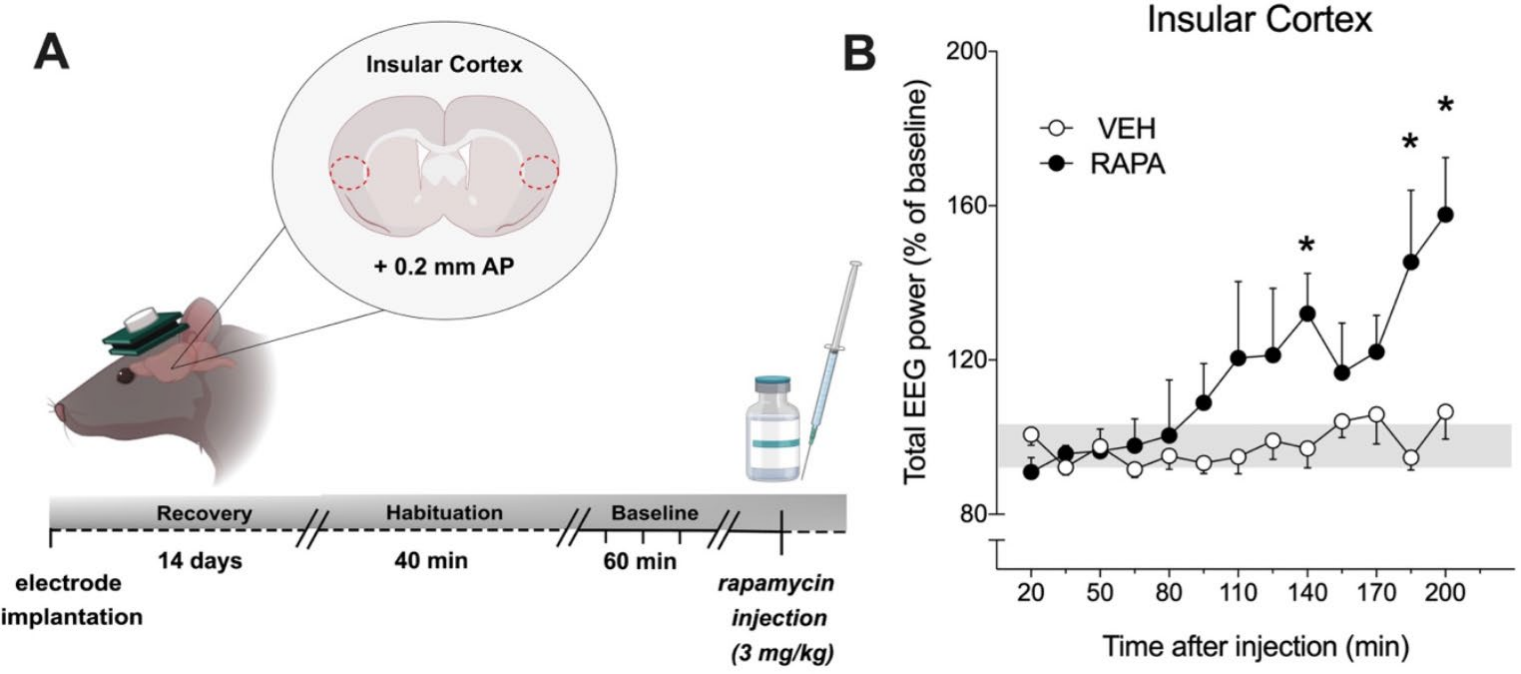
Schematic design and results of *experiment 1.***(A)** Schematic representation of the study design of *experiment 1* analyzing neuronal activity in the insular cortex following acute treatment with rapamycin. **(B)** EEG signals (expressed as percentage of baseline EEG activity) were telemetrically recorded before and after single i.p. injection of rapamycin (RAPA, 3 mg/kg). Data are expressed as means ± SEM (RAPA: *n* = 7; vehicle VEH: *n* = 9; *p < 0.05 compared to vehicle

**Fig. 2:**
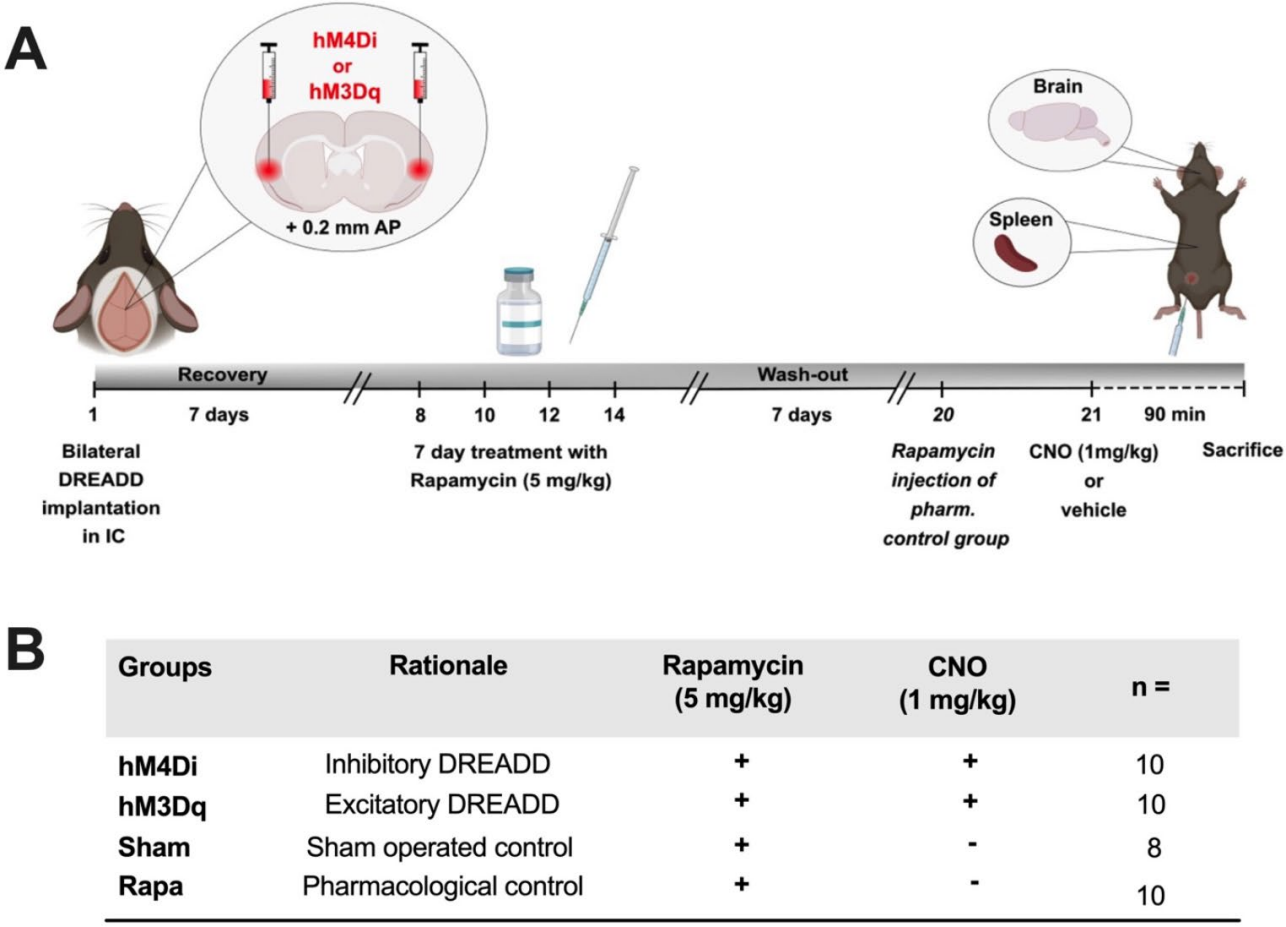
Schematic design of *experiment 2.***(A)** Schematic representation of the study design and group allocation. Rats were injected with an AAV vector resulting in CaMKII-driven expression of *hM4D(G)i*- or *hM3D(Gq)-*mCherry in the IC. **(B)** Subsequently, animals were randomly assigned to the experimental and control groups and subjected to a 7 day-schedule of pharmacological treatment with a therapeutically relevant dose of the small-molecule immunosuppressive drug rapamycin (5 mg/kg). After a drug wash-out period of 7 days, inhibitory and excitatory DREADDs were activated by i.p. injection of CNO (1 mg/kg). Animals were sacrificed 90 min post CNO injection and spleens and brains were collected to analyze the peripheral immune status as well as DREADD location and functioning

The present study consisted of two complementary experiments. In *experiment 1*, neuronal activity was measured in the IC by electroencephalography (EEG) for 200 min following administration of an acute moderate dose (3 mg/kg) of rapamycin (Fig. 1A). Subsequently, *Experiment 2* was conducted to determine whether a previously experienced period of pharmacologically induced immunosuppression may be reinstated by nonspecifically manipulating neuronal activity in the IC. For this purpose, rats were bilaterally injected with either inhibitory *hM4Di-* or excitatory *hM3Dq* AAV-CaMKIIa-DREADDs (Designer Receptors Exclusively Activated by Designer Drugs) before being subjected to a 7 day-schedule of pharmacological treatment with a high dose of rapamycin (5 mg/kg). After a drug wash-out period of 10 days, DREADDS were activated by i.p. injections with the inert ligand clozapine *N*-oxide (CNO). Rat spleens and brains were harvested 90 min post CNO injection. This time point was chosen based on a previous study, verifying pronounced conditioned immunopharmacological effects as well as plasma concentrations starting to peak at around 60 min following rapamycin administration (Baker et al. 1978; Zimmerman and Kahan 1997; Lückemann et al. 2019). The peripheral immune status was compared with animals acutely treated with rapamycin (5 mg/kg), DREADD location and functioning were verified by immunohistology (Fig. 2A). A subsequent pharmacological experiment aimed at analyzing the impact of CNO on splenic cytokine production. A group of animals received a single administration of CNO (1 mg/kg) and cytokine production of ex vivo stimulated splenocytes was compared with vehicle (VEH)-treated animals 90 min following injection.

### Drugs and Viral Constructs

The applied drug doses of rapamycin were chosen based on previous studies proving therapeutic efficacy in several animal disease models (Alain et al. 2010; Pech et al. 2011; Huang et al. 2012; Hetze et al. 2022) and taste-immmune associative learning studies (Lückemann et al. 2019, 2021). Briefly, the TORC1 inhibitor rapamycin (LC Laboratories, Woburn, MA, USA) was dissolved in ethanol (750 µL) and cremophor (1500 µL). This stock solution was further diluted with sterile saline according to the animal’s individual weight to achieved the desired dose of 5 mg/kg with a final injection volume of 0.5 mL. CNO was dissolved in DMSO (final concentration: 2.5%) and further diluted with sterile saline to gain the injection dose of 1 mg/kg. Both, rapamycin and CNO were administered intraperitoneally (i.p.). Ca^2+^/calmodulin-dependent protein kinase IIa (CaMKIIa)- promoter driven adeno-associated viral vectors AAV-CaMKIIa-hM4D(Gi)-mCherry (Addgene plasmid # 50,477; http://n2t.net/ addgene:50,477; RRID: Addgene_50477; titer ≥ 1 × 10^13^ vg/mL) and AAV-CaMKIIa-hM3D(Gq)-mCherry (Addgene plasmid # 50,476; http://n2t.net/addgene: 50,476 ; RRID: Addgene_50476; titer ≥ 1 × 10^13^ vg/mL) were used to transduce projection neurons with hM4Di or hM3Dq, respectively. An mCherrry reporter allowed for visualization of expression.

### Intracerebral EEG Recordings

In *experiment 1* electrode implantation and EEG recordings were performed as described previously with modifications (Engler et al. 2011; Hadamitzky et al. 2014). Briefly, animals were deeply anaesthetized with a mixture of ketamine hydrochloride (90 mg/kg; Ketanest, Parke-Davis, Karlsruhe, Germany) and xylazine hydrochloride (15 mg/kg; Rompun, Bayer Health Care, Leverkusen, Germany). Monopolar stainless steel EEG electrodes (outer diameter: 0.25 mm, insulated with polyurethane except for the tip) were stereotactically implanted into the left or right IC of anesthetized animals (distribution was equally over both sites). The coordinates used for EEG recording were as follows: antero-posterior (AP) + 0.2 mm; medio-lateral (ML) ± 5 mm; dorso-ventral (DV) -6.0 mm relative to bregma (Paxinos and Watson 1998). As indifferent electrode, a stainless-steel screw was positioned at the surface of the cerebellum. The electrodes were soldered to a socket (TSE Systems, Bad Homburg, Germany) and fixed to the skull with dental cement (Technovit 3040, Heraeus Kulzer, Wehrheim, Germany). For postoperative care rats were treated with antibiotic (Retacillin compositum, 200.000 IE, i.m.; mibe GmbH Arzneimittel, Brehna, Germany) and analgesic (Rimadyl^®^, 5 mg/kg, s.c.; Pfizer, USA), and were allowed to recover from surgery for 14 days. On testing days, animals were transferred to the experimental room and habituated to the new environment for at least one hour. EEG transmitters (TSE Systems) were plugged to the implanted electrode socket and baseline EEG activity (three 5 min blocks separated by 10 min intervals) was recorded from the freely moving rats. Subsequently, animals were gently lifted and injected with rapamycin or vehicle and EEGs were recorded for 200 min. EEG signals were telemetrically received via pulse position modulation, transmitted to a decoder, and digitized with a sample rate of 128/s. After removing artifacts from the recordings, power density spectra were computed for periods of 4 s by fast Fourier transformation and averaged for each 5 min block (Dimpfel et al. 1986). Absolute EEG power (µV) of the recorded frequency range (0.6–30 Hz) was summarized and mean total EEG power was calculated (Pacheco-López et al. 2013; Prager et al. 2013; Hadamitzky et al. 2014). Data are expressed as percentage of baseline EEG activity. The correct placement of the electrodes was verified histologically at the end of the experiment.

### Viral Injection

Animals were anaesthetized and surgically handled as described above. After skin incision, a hand drill was used to make two holes above the posterior IC: AP +0.2 mm; ML ± 5 mm relative to bregma (Paxinos and Watson 1998). A custom-made glass pipette attached to a syringe was lowered − 6 mm DV to the IC and 0.3 µL plasmid was injected bilaterally using pressure injection. The pipette was left in place for an additional 5 min to allow for diffusion before it was retracted slowly. Bone wax (Coherent Scientific) was placed over the drilled holes and the skin was sutured. Following surgery, animals were allowed to recover for one week while receiving post-operative care (analgesic: carprofen, 5 mg/kg s.c.) for three days.

### Splenic Cytokine Production

Spleens were disrupted with a syringe plunger in a Petri-dish containing cold HBSS (1 x Hank’s Balanced Salt Solution, gibco^®^, Life Technologies™, Darmstadt, Germany). Isolated splenocytes were transferred into Falcon tubes and erythrocytes were lysed with BD Pharm Lyse™ solution (BD Pharmingen, Heidelberg, Germany). Splenocytes were then washed in cell culture medium (RPMI, 10% FBS, 50 µg/mL gentamycin) and filtered through a 70 μm nylon cell strainer. Cell concentrations were determined with an automatic animal cell counter (Vet abc; Medical Solution, Steinhausen, Switzerland) and adjusted to a final concentration of 5 × 10^6^ cells/mL. To stimulate cytokine production, splenocytes were incubated with 50 ng/mL phorbol 12-myristate 13-acetate (PMA; Sigma Aldrich) and 500 ng/mL ionomycin (Sigma Aldrich) for 24 h at 37° C and 5% CO_2_. Cytokine concentration in cell culture supernatants was measured by electrochemiluminescence using a V-PLEX Proinflammatory Panel 2 Rat Kit on a MESO QuickPlex SQ 120MM instrument (Meso Scale Discovery, Rokville, MD, USA) according to manufacturer´s instructions.

### Brain Preparation and Immunofluorescence Staining

The brains were immersed in 4% paraformaldehyde (PFA) for 48 h followed by 30% sucrose immersion for at least 72 h, until saturated. IC coronal sections of 40 μm were cut on a cryostat (Leica CM1950, Leica Biosystems, Wetzlar, Germany) and were collected in 6 well cell culture plates (TPP Techno Plastic Products AG, Trasadingen, Switzerland) containing PBS. The sections were stored in cryoprotective solution at -20° C until further use. All staining steps described were performed free-floating. To remove excess embedding material and cryoprotective solution, all brain sections were washed in PBS (3 × 5 min) before proceeding with the respective staining protocols.

For c-Fos staining, brain sections underwent a heat-induced epitope retrieval (HIER) in sodium citrate buffer (10 mM sodium citrate, 0.05% Tween^®^ 20 (v/v, AppliChem, Darmstadt, Germany), pH 6.0) for 20 min at 85° C. After a cooling time of 15 min at room temperature (RT), sections were then washed again in PBS (3 × 5 min) and blocked in 10% normal goat serum (NGS, v/v, Biozol, Echingen, Germany) and 20% avidin solution (v/v, Vector Laboratories, Newark, CA, USA) in 0.2% PBS-Triton^®^X-100 (PBS-Tx, v/v, Sigma Aldrich, Steinheim, Germany) for 90 min at RT to reduce nonspecific antibody binding. Subsequently, tissue sections were incubated with rabbit anti c-Fos antibody (1:500, cat. No. 226 003, Synaptic Systems, Göttingen, Germany), 1% NGS and 20% biotin solution in 0.2% PBS-Tx overnight at RT. The following day, tissue sections were washed in PBS (3 × 10 min) and subsequently incubated with biotinylated goat anti-rabbit IgG antibody (1:500, cat. No. BA-1000, Biozol, Eching, Germany) and 1% NGS in 0.1% PBS-Tx for 90 min at RT. Brain sections were then washed again in PBS and incubated with Alexa Fluor 488-conjugated streptavidin (1:1000, cat. No. 016-540-084, Biozol, Eching, Germany) and 1% NGS in 0.1% PBS-Tx for 90 min at RT. Glutamate decarboxylase (GAD) 67 staining was performed by blocking rinsed sections in 10% NGS in PBS for 90 min at RT. Afterwards, sections were incubated with a primary antibody solution (mouse anti GAD67 1:500, cat. No. MAB5406, Merck Millipore, Burlington, MA, USA) containing 1% NGS in PBS overnight at 4°C. The slices were again rinsed with PBS (3 × 10 min) before being incubated with a secondary antibody solution (goat anti-mouse IgG AF488 1:500, cat. No. 1038-30, Biozol, Eching, Germany) containing 1% NGS in PBS for 90 min at RT. For parvalbumin (Parv) staining, rinsed sections were incubated in 10 mM sodium citrate buffer (pH 6.0) with 0.05% Tween^®^ 20 for 20 min at 85°C. Following a cooling time of 15 min at RT, sections were then washed again in PBS (3 × 5 min) and blocked in 10% NGS in 0.2% PBS-Tx for 90 min at RT. Subsequently, sections were incubated with a primary antibody solution (mouse anti parvalbumin 1:1000, cat. No. 195 011, Synaptic Systems, Göttingen, Germany) containing 1% NGS in 0.2% PBS-Tx overnight at RT. The following day, the slices were washed in PBS (3 × 10 min) and incubated with a secondary antibody solution (goat anti-mouse IgG AF488 1:500, cat. No. 1038-30, Biozol, Eching, Germany) containing 1% NGS in 0.1% PBS-Tx for 90 min at RT. After the incubation periods with the respective fluorescent antibodies, sections were washed in PBS and stained with (1 µg/mL) 4’,6-Diamidino-2-phenylindol (DAPI; Carl Roth, Karlsruhe, Germany) for another 10 min. Finally, sections were washed in PBS again before placing them on microscope glass slides (SuperFrost™ Microscope Slides; cut; Thermo Fisher Scientific) and mounting them with immunoselect antifading mounting medium (SCR-038447, Dianova by BIOZOL, Eching, Germany).

### Immunofluorescence Imaging and Quantification

A Zeiss Axio Scan Z.1 microscope (Carl Zeiss, Oberkochen, Germany) with ZEN Blue software 3.6 was used for image collection. For each animal, six whole-section images were taken with fixed settings at 10 x magnification. All images were processed with Fiji software (Fiji is Just ImageJ; version: 2.9.0/1.53t; National Institutes of Health) and cell counting was performed semi-automatically with the Fiji ‘thresholding’ and ‘analyze particles’ tools by an experimenter blinded to the treatment. Prior to quantification, whole-section images were converted into grayscale to provide clearer contrast between c-Fos expression and basal expression. The location of IC was determined by comparison with rat brain atlas and manually assigned as a region of interest (ROI) using the ‘rectangle selection’ tool. For each animal and tissue section, an equal-sized rectangle was used as ROI. The size and corresponding coordinates of the ROIs were stored in an excel spreadsheet for each individual brain section. c-Fos immunoreactive cells were counted in six separate ROIs per animal from both hemispheres and averaged. For this purpose, the triangle algorithm was applied for thresholding. Only animals with bilateral fluorescent signals within the area of the IC were included. One animal of the hM3Dq group had to be excluded from the analyses due to failure of DREADD expression. c-Fos immunoreactive cells were identified as fluorescent oval-shaped nuclei with a particle size of 50–500 µm and a circularity of 0.3-1.00. Autofluorescent artifacts that were incorrectly counted as c-Fos immunoreactive cells were manually excluded. The averaged c-Fos count was related to the total area of the ROI (= region of the IC). Additionally, the area of DREADD expression within the respective ROIs was determined. For this purpose, the boundaries of DREADD expansion were manually outlined using the Fiji ‘polygon selection’ tool and saved as ROIs (DREADD ROI). The boundaries of the DREADD area were defined to the point where both the mCherry signal, as well as DREADD labeled neurons were clearly evident. The area of the selected DREADD ROIs and thus the DREADD expansion was determined with the Fiji ‘measuring’ tool and averaged. c-Fos^+^ labeled cells were recounted within the respective DREADD ROIs and the averaged cell count was related to the averaged area of DREADD expansion.

### Statistical Analysis

Statistical analyses were performed with Sigma Plot (Version 12.3, Systat Software San Jose, CA, USA) and the level of significance was set at *p* < 0.05. Normality of residuals was examined using the Shapiro-Wilk test, since this test has a high statistical power, especially for sample sizes < *n* = 50 (Razali 2011). Cytokine production and c-Fos expression were analyzed using one-way ANOVA. Tukey-corrected post hoc comparisons were made to compare groups on individual sessions. All comparisons were two tailed. Data in figures are expressed as the mean ± SEM. Data are shown and evaluated as mean percentage changes from Sham controls.

## Results

### Intracerebral EEG Recordings

In *experiment 1* rats’ total EEG baseline power in the IC did not differ between brain sides and treatment groups, respectively (saline: 5792 ± 221 µV, rapamycin: 4178 ± 52 µV). Acute systemic administration of rapamycin resulted in a marked alteration in total EEG power in the IC (Fig. 1B). Repeated measures ANOVA revealed a significant “treatment” effect between saline and rapamycin groups in the IC (F(1,168) = 4.91, *p* < 0.044), an effect for the factor “time” (F(12,168) = 5.264, *p* < 0.001), as well as a significant “treatment x time” interaction (F(12,168) = 3.53, *p* < 0.001). Independent t-tests revealed significant increases of EEG power in the IC at 140, 185, and 200 min (*p* ≤ 0.05) after peripheral injection of rapamycin compared to saline treated animals.

### DREADD Location and Validation

**Fig. 3:**
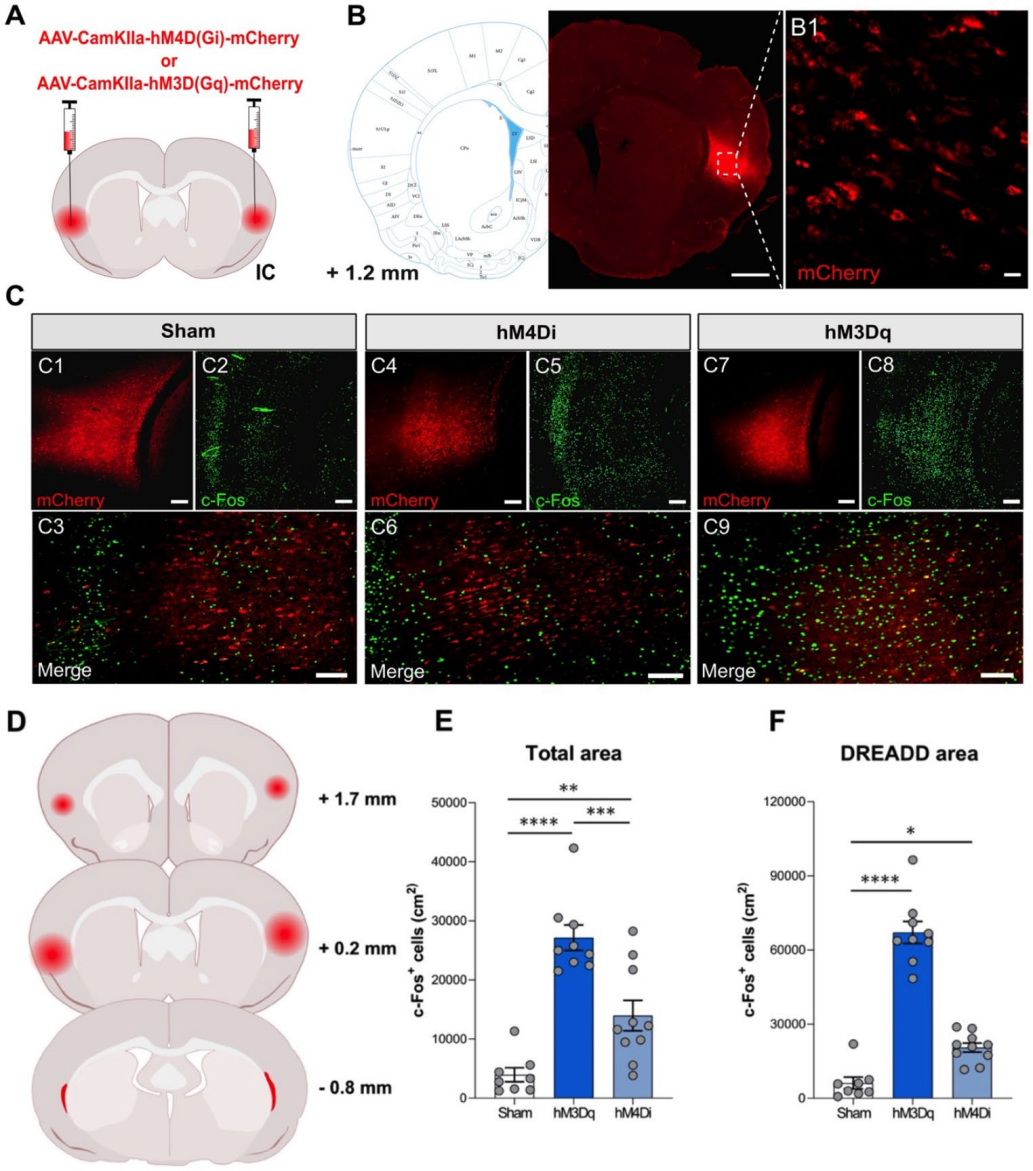
Location and validation of DREADDs in the insular cortex (IC). (**A**) Schematic representation of the bilateral injection sites of either inhibitory AAV-CaMKIIa-hM4D(Gi)-mCherry or AAV-CaMKIIa-hM3Dq-mCherry. (**B**) Representative overview of DREADD location and expression (shown in red) in the insular cortex. Scale bar: 1000 μm. (**B1**) DREADD expression (shown in red) in cell bodies and fibers of cortical neurons in the IC. Scale bar: 20 μm. (**C**) Representative images of c-Fos expression after DREADD activation with CNO (DREADD = red; c-Fos = green). (**C1-C3**) c-Fos expression in sham animals receiving a vehicle saline solution 90 min prior to sacrifice. Scale bars: C1 & C2 = 200 μm, C3 = 100 μm. (**C4-C6**) c-Fos expression in the *hM4Di* DREADD group following DREADD activation with 1 mg/kg CNO (i.p.). Scale bars: C4 & C5 = 200 μm, C6 = 100 μm. (**C7-C9**) c-Fos expression in *hM3Dq* DREADD group following DREADD activation with 1 mg/kg CNO (i.p.). Scale bars: C7 & C8: 200 μm, C9: 100 μm. (**D**) Coronal reconstruction of *hM3Dq* and *hM4Di* DREADD expansion and expression throughout the IC. (**E**) Quantification of c-Fos expression normalized to the total area of the IC after DREADD activation with CNO. Asterisks represent statistically significant differences between experimental groups (ANOVA; Tukey t-test ***p < 0.001. hM3Dq = activating DREADD + CNO; hM4Di = inhibitory DREADD + CNO; Sham = hM3Dq or hM4Di DREADD + saline vehicle). All data are means ± SEM and shown as the total cell count of c-Fos^+^ per cm^2^. (**F**) Quantification of c-Fos expression normalized to the DREADD area within the IC after DREADD activation with CNO. Asterisks indicate statistically significant differences between experimental groups (ANOVA; Tukey t-test *p < 0.05, ***p < 0.001. hM3Dq = activating DREADD + CNO; hM4Di = inhibitory DREADD + CNO; Sham = hM3Dq or hM4Di DREADD + saline vehicle). All data are means ± SEM and shown as the total c-Fos^+^ cell count per cm^2^; *n* = 8–10/group)

Accuracy of stereotaxic coordinates as well as the surgical procedure was verified with the rat brain atlas according to Paxinos and Watson (1998). The observed mCherry expression was restricted to the IC with no evidence of DREADD transduction or retrograde transport into other brain regions. Moreover, fluorescent signals were primarily situated in cell bodies of cortical neurons and the respective neuronal fibers throughout the injected area. DREADD expansion in the IC ranged from + 1.7 mm to -0.8 mm relative to bregma with the greatest expression around the injection site at + 1.2 mm (Fig. 3D). No differences in virus expansion throughout the IC were seen between inhibitory *hM4Di* and excitatory *hM3Dq* groups.

DREADD validation upon activation with CNO was examined by c-Fos staining and quantification (Fig. 3C). In the *Sham* control group, histology showed basal c-Fos expression present within the entire cortex, including the IC (Fig. 3C**1-C3**). In comparison, the *hM4Di* group displayed a specific pattern of c-Fos expression with enhanced activity surrounding the axial mCherry signal and minimal c-Fos within the area of highest DREADD expression (Fig. 3C**4-C6**). Conversely, pronounced expression of c-Fos was observed in the activating *hM3Dq* group, which distinctly colocalized with the area of DREADD expression (Fig. 3C**7-C9**). When quantified in the total IC area (in cm^2^), statistical analysis revealed an effect for normalized counts of c-Fos expression (ANOVA; F(2,24) = 27.64, *p* < 0.0001; Fig. 3E). Post-hoc testing indicated that DREADD activation resulted in significantly increased c-Fos expression in both the *hM3Dq* (*p* < 0.0001) and the *hM4Di* (*p* < 0.0089) groups compared to *Sham* controls. Moreover, c-Fos expression in the *hM3Dq* group was significantly higher compared to the *hM4Di* group (*p* < 0.0005). Statistical analysis also revealed an effect for c-Fos expression normalized to the respective DREADD expansion area (ANOVA; F(2,24) = 101.8, *p* < 0.0001; Fig. 3F). Likewise, post-hoc testing showed that DREADD activation led to increased c-Fos expression within the *hM3Dq* (*p* < 0.0001) and *hM4Di* (*p* < 0.0095) groups compared to *Sham* controls. As seen for the total IC area, c-Fos expression of the *hM3Dq* group was significantly elevated in the respective DREADD expansion area compared to the *hM4Di* group (*p* < 0.0001; Fig. 3F). Further Immunofluorescent labeling revealed co-localization of AAV-CaMKIIa-hM4D(Gi)-mCherry with GAD67^+^ and Parv^+^ neurons in the IC (**Supplementary Fig. 2**).

To indirectly monitor efficacy of rapamycin, the animal’s body weight was assessed before and after the seven-day treatment regimen. Statistical analyzes revealed a significant effect on weight gain during this time (ANOVA; F(30,160) = 21.628; *p* < 0.001; **Supplementary Fig. 1**). While the untreated pharmacological control group (*Rapa*) showed an increase in body weight over the seven days, the daily treated experimental groups (*hM3Dq*,* hM4Di*,* Sham*) displayed either maintenance or a slight decrease in body weight compared to the beginning of treatment (*p* < 0.05).

### Splenic Cytokine Production after Chemogenetic Manipulation of Insular Cortex Activity

**Fig. 4:**
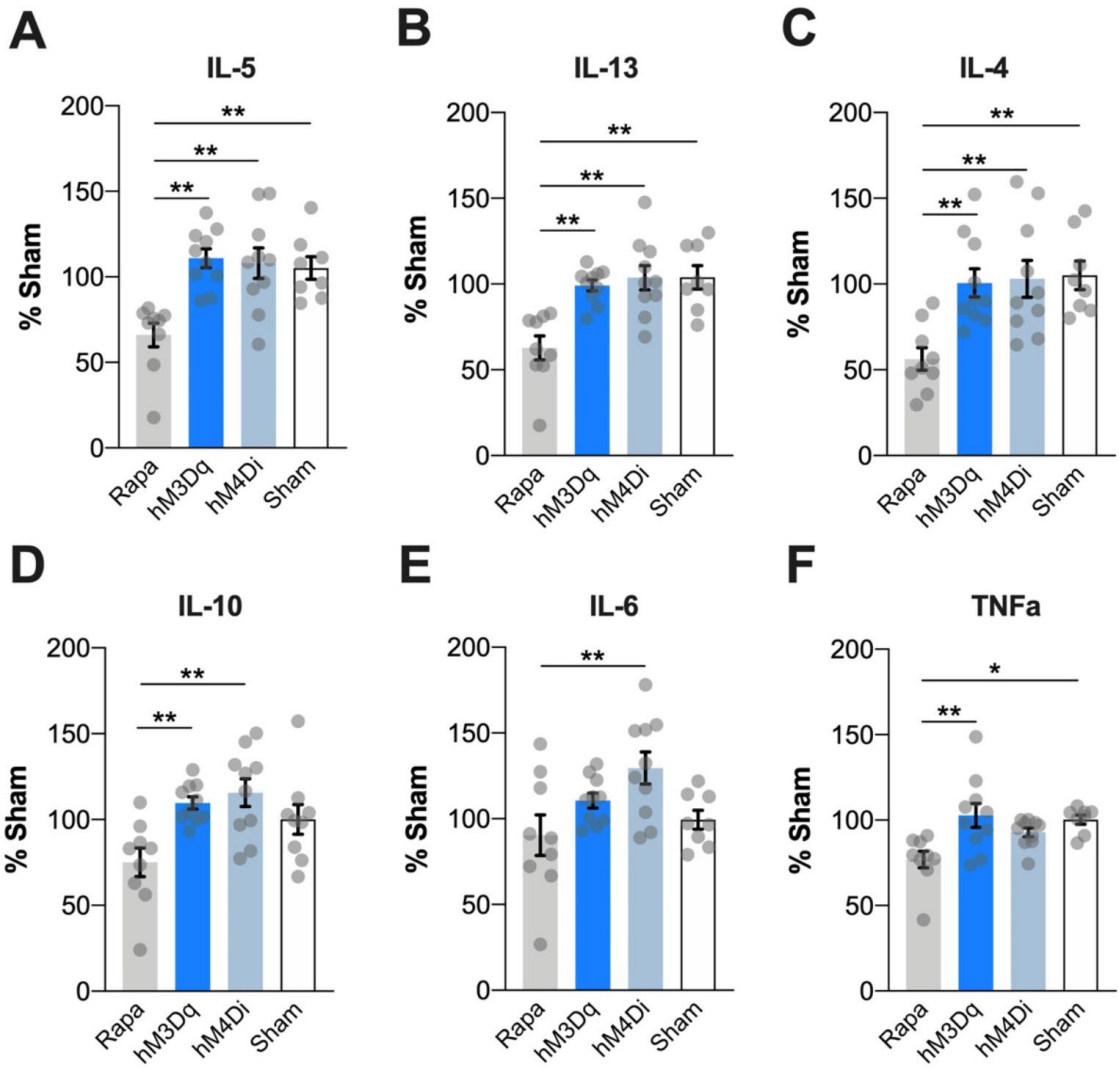
Splenic cytokine levels after chemogenetic manipulation of insular cortex activity. 90 min following DREADD activation, splenocytes were isolated and stimulated with 50 ng/ml PMA and 500 ng/ml Iono before cytokine production was measured in the supernatants using MSD multiplex assay. Asterisks represent a statistically significant difference between experimental groups (ANOVA; Tukey t-test *p < 0.05, **p < 0.01, ***p < 0.001; *Rapa* = pharmacological control group (5 mg/kg); *hM3Dq* = activating DREADD + CNO; *hM4Di* = inhibitory DREADD + CNO; Sham = *hM3Dq* or *hM4Di* DREADD + vehicle; *n* = 8–10/group). All data are means ± SEM and shown as percentage changes of *Sham* controls

Cytokine analyses in culture supernatants of ex vivo stimulated splenocytes following manipulation of the IC revealed effects of treatment on IL-5 (ANOVA; (F(3,33) = 8.703; *p* < 0.0002; Fig. 4A), IL-13 (F(3,33) = 10.20; *p* < 0.0001; Fig. 4B), IL-4 (F(3,33) = 6.919; *p* < 0.0010; Fig. 4C), IL-10 ( (F(3,34) = 5.809; *p* < 0.0026; Fig. 4D), IL-6 (F(3,33) = 4.176 *p* < 0.0130; Fig. 4E), and TNFα (F(3,33) = 5.714 *p* < 0.0029; Fig. 4F) cytokine production. Post-hoc testing showed that overall cytokine production was only significantly reduced in the group, acutely treated with rapamycin *(Rapa)* compared to either *hM3Dq-*, *hM4Di-*groups, or *Sham* animals (*p* < 0.05).

To assess potential off-target effects of CNO, masking or compensating potentially reinstated immunosuppressive effects of rapamycin, a subsequent experiment analyzed the impact of CNO on peripheral immune functions. Compared to vehicle (VEH)-treated animals, single administration of CNO (1 mg/kg) did not alter cytokine production of ex vivo stimulated splenocytes (**Supplementary Fig. 3**; *n* = 6/group; Unpaired t-test).

## Discussion

The current study was designed to investigate the ability of the IC to process states of pharmacologically induced immunosuppression. For one, neuronal activity in the IC was measured by electroencephalography (EEG) following an acute injection with a moderate dose (3 mg/kg) of the mTOR inhibitor rapamycin. For another, it was determined whether a previously experienced period of pharmacologically induced immunosuppression may be reinstated by nonspecifically manipulating neuronal activity in the IC using either inhibitory *hM4Di-* or excitatory *hM3Dq* AAV-CaMKIIa-DREADDs.

The view that the CNS and the immune system communicate through complex bidirectional pathways was already proposed a long time ago, hypothesizing that the immune system operates as a sensory organ which informs the CNS about infection and injury (Besedovsky and Sorkin 1977; Blalock 1984). Amongst others, this concept of a sensing process was confirmed by data documenting altered dynamics of electrical activity not only in the IC but also in the amygdala following acute challenges with antigens such as lipopolysaccharide or staphylococcal enterotoxin B (Doenlen et al. 2011). The observation of increased electrical activity recorded from the IC via deep-brain EEG within the first 200 min after acute low dose treatment with rapamycin extends this concept of a sensing process to states of immunosuppression. However, following a previously experienced period of pharmacological immunosuppression, though, diminished splenic cytokine production as induced by rapamycin could not be reinstated by nonspecific neuronal activation or inhibition of this brain structure. This allegedly failure on immune functions is unlikely to be explained simply by poor perception of a suppressed immune status due to deficient pharmacological action of rapamycin. Effectiveness of the drug was indirectly reflected by maintenance or reduction of the animal’s body weight. The mTOR pathway, in particular the target complex TORC1, is strongly involved in the regulation metabolic processes (Li et al. 2014; Szwed et al. 2021) also reflected by reduced food intake and body weight gain in free-feeding rats receiving rapamycin (Cambiaghi et al. 2013; Hadamitzky et al. 2018).

CNO or clozapine have primarily involved schizophrenic patients during therapy, leaving uncertainties about the impact on rodent immune functioning. Evidence revealed that CNO can be back-metabolized to pharmacologically active clozapine, and both compounds have frequently been suggested to exert modulatory effects on peripheral immunity even though evidence is lacking. However, we here show that single CNO treatment did not alter affect splenic cytokine production in rats, clearly ruling out off-target effects of CNO that may have masked or compensated immunosuppressive effects of rapamycin.

Malfunctioning or incorrect DREADD placement as cause for the absence of peripheral immunological alterations can be ruled out as well. Consistent with current literature, activation of *hM3Dq*-DREADDs via CNO resulted in significantly elevated c-Fos^+^ cell counts, indicative for enhanced neuronal excitability (Scarlata et al. 2019). The observation that c-Fos expression distinctly colocalized with the fluorescent mCherry signal clearly indicates that the elevated cell counts are attributable to the activation of *hM3Dq*-DREADDs. However, contrary to the anticipated outcome that activation of inhibitory *hM4Di*-DREADDs leads to silencing or attenuation of neuronal excitability within the DREADD area, its activation also induced to elevated c-Fos^+^ cell counts compared to *Sham* controls. This seemingly paradoxical finding has already been reported with CaMKIIa-driven inhibitory DREADDs (López et al. 2016; Bubb et al. 2020). One possible explanation refers to the scant cellular specificity of the CaMKIIa promotor. Its expression was initially suggested to only occur within principal excitatory projection neurons of the mammalian cortex. However, coherent to ample evidence (Liu and Murray 2012; Haery et al. 2019; Keaveney et al. 2020; Radhiyanti et al. 2021; Veres et al. 2023), the observed co-localization of *hM4Di*-mCherry and *GAD67* or *Parv* points out that this promotor also drives DREADD expression in gamma-aminobutyric acid (GABA)-ergic interneurons. More specific, viral serotype AAV9 constructs driven by CaMKIIa have not only been shown to infect *Parv*, somatostatin (*SST*), cholecystokinin (*CCK)* and neuropeptide Y (*NPY*) expressing interneurons, but also to excite them (Watakabe et al. 2015; Keaveney et al. 2020; Radhiyanti et al. 2021; Veres et al. 2023). Thus, due to apparent silencing of several infected inhibitory neurons, neuronal disinhibition may have occurred, culminating in enhanced activity of surrounding DREADD-negative neurons (Smith et al. 2016). This hypothesis is further fostered by evidence revealing that chemogenetic inhibition of cortical *Parv* interneurons culminated in elevated cortical excitation (Markicevic et al. 2020; Yiannakas et al. 2021). Disinhibition may also result from changes in local circuits or even larger networks involving feedback mechanisms from different in- or output regions (Goossens et al. 2021). Accordingly, the silencing of excitatory CaMKIIa^+^ neurons has potentially been masked by compensatory effects.

The IC is one of the most intricate anatomical hubs in the mammalian brain. It’s extensive connections to other brain regions comprise strong projections to the striatum and reciprocal connections with various thalamic and amygdaloid sub-regions (Haaranen et al. 2020). Noteworthy, peripheral administration of rapamycin not only affects neuronal activity in the IC, but also in the amygdala (Hadamitzky et al. 2014). In turn, both, the amygdala and the IC receive strong projections from the nucleus of the solitary tract (Kawai 2018; Fermin et al. 2022; Zheng et al. 2022; Wang et al. 2023), which processes vagal signals that are relayed to other brain regions across various neuronal circuits (Pavlov and Tracey 2017). Thus, if the information of an experienced status of pharmacologically induced immunosuppression is represented in the brain, it may not solely be in the IC.

Recent work revealed that during disease stages of colitis and peritonitis active neuronal IC ensembles were captured using activity-dependent cell labeling in mice (FosTRAP). Importantly, chemogenetic reactivation of these neuronal ensembles was sufficient to retrieve the inflammatory state under which these neurons were captured (Koren et al. 2021). These findings suggest that the information of peripheral inflammatory conditions is obviously captured as a specific neuronal trace or engram and thus physically represented as memory in the IC (Koren and Rolls 2022). The formation of such a trace, also termed as “immunengram”, comprises changes in both neuronal circuits and peripheral tissue components. From an evolutionary perspective, though, this concepts makes sense since it enables the brain not only to better communicate with the immune system but also to precisely orchestrate adequate physiological processes in peripheral tissues (Rolls 2023). Contrary, placebo studies have shown that experienced drug effects or mere expectation are not sufficient to reinstate immunopharmacological drug effects (Albring et al. 2012). Placebo responses, however, can be induced via taste-immune associative learning. Such approaches are based on classical conditioning of physiological responses on the one hand and the bidirectional communication between the CNS and the immune system on the other hand (Irwin and Cole 2011; Pavlov et al. 2018). Most commonly, a novel taste stimulus (conditioned stimulus/CS) is paired with the administration of an immunopharmacological agent (unconditioned stimulus/US). After one or several CS-US pairings, the sole presentation of the CS elicits a conditioned response which qualitatively resembles the drugs’ pharmacological effects (Hadamitzky et al. 2020; Bouton et al. 2021). Importantly, the phenomenon of taste-immune associative learning has been shown to be mediated via a basic neural circuit comprising sensory and hedonic pathways with the IC being essentially involved in the integration of gustatory and visceral stimuli (Sewards and Sewards 2001; Sewards 2004). It has also been revealed that conditioned suppression of splenocyte proliferation and cytokine production (IL-2 and IFN-γ) was affected by excitotoxic IC lesioning(Ramirez-Amaya et al. 1996; Ramirez-Amaya and Bermudez-Rattoni 1999; Pacheco-Lopez et al. 2005). These findings indicate that the IC is capable of forming representations of immunosuppressive effects but only via taste-associative learning and memory.

The ability of the brain to form a central representation of the organism’s immunological state e.g., following infection or exposures to allergens, toxins, or antigens holds high adaptive value. By constantly monitoring changes in immune activity the brain is also able to regulate the immune system for generating appropriate physiologically synchronized responses (Rolls 2023). Also, the conditioning of immune functions has very likely evolved evolutionary over the years as an adaptive, associative learning mechanism to protect the organism from potentially harmful immune responses that occurred after contact or ingestion of immunomodulating substances (e.g. toxic fruits) (Ader 2003; Bermudez-Rattoni 2004; Hadamitzky et al. 2020). Despite the fact that the IC perceived changes in the organisms’ immune homeostasis caused by the mTOR inhibitor rapamycin, the concept of a formed “immunengram” does apparently not apply for drug induced stages of immunosuppression. However, this finding does make sense since most immunosuppressive agents or drugs were only discovered or synthetized 60 years ago. Thus, there was no necessity or time for the organism and especially the brain to form images of an altered immune homeostasis induced by artificial compounds.

### Limitations and Outlook

When discussing the experimental outcome, it is noteworthy to address the anatomical organization of the IC and its role in the perception and retrieval of immune-related information. The IC comprises two main sub-regions, differing in cytoarchitecture and functional properties (Haaranen et al. 2020; Mathiasen et al. 2023). The anterior IC (aIC) is strongly connected to the amygdala and putatively involved in processing emotions, empathy, social cognition and decision making. The posterior IC (pIC) receives more input from sensory cortices and is associated with the processing of visceral somatosensory information (Gehrlach et al. 2020; Rolls 2023). Accordingly, the pIC has been hypothesized to be the primary interoceptive cortex (Appenzeller et al. 2022), thus, serving as the target region for chemogenetic manipulations in the present study. However, evidence indicates that both sub-regions are engaged in interoceptive processes and therefore potentially involved in “immunoception” (Gogolla 2017; Wu et al. 2020; Berntson and Khalsa 2021; Chen et al. 2021). Consequently, the interaction of both sub-regions may be critical for storing and remembering immunosuppression-related information. Given this background, it is at least conceivable that the additional inclusion of the aIC in the DREADD-mediated manipulation of neuronal activity may have been more informative regarding putatively representations of immunosuppressive effects in the IC.

The IC is suggested to exhibit functional lateralization, while the left-side is rather associated with parasympathetic homeostatic afferents as well as behaviors such as feeding, safety, positive affect and approach behaviors. The right-sided IC is proposed to be predominantly associated with sympathetic nervous system responses with an important role in perception and processing of immune-associated information (Montalbano and Tubbs 2018; Koren and Rolls 2022). Thus, it cannot be excluded that the bilateral manipulation of the IC culminated in compensatory effects emanating from the left IC or its associated circuits, potentially affecting the acquired outcome.

## Electronic Supplementary Material

Below is the link to the electronic supplementary material.

Supplementary Material 1

## Data Availability

No datasets were generated or analyzed during the current study.
