# Supplementary material for: Investigations on the Ability of the Insular Cortex to Process Peripheral Immunosuppression
Source: J Neuroimmune Pharmacol. 2024 Jul 30;19(1):40. doi: 10.1007/s11481-024-10143-9 (PMC11289148; doi:10.1007/s11481-024-10143-9)
Supplement: Supplementary file 1 — Supplementary Material 1 [file 11481_2024_10143_MOESM1_ESM.docx]

***Appendix A. Supplementary data***

**Investigations on the ability of the insular cortex to process peripheral immunosuppression**

**Authors:**

Julia Bihorac^1^, Yasmin Salem^1^, Laura Lückemann^1^, Manfred Schedlowski^1,2^, Raphael Doenlen^3^, , Harald Engler^1^, Melanie D. Mark^4^, Kirsten Dombrowski^1^, Katharina Spoida^5^, Martin Hadamitzky^1*^

**Affiliations:**

^1^Institute of Medical Psychology and Behavioral Immunobiology, Center for Translational Neuro- Behavioral Sciences (C-TNBS), University Hospital Essen, University of Duisburg-Essen, Essen, Germany

^2^Department of Clinical Neuroscience, Osher Center for Integrative Medicine, Karolinska Institutet, Stockholm, Sweden

^3^Center of Phenogenomics, School of Life Sciences, Ecole Polytechnique Fédérale de Lausanne, Lausanne, Switzerland

^4^Behavioral Neuroscience, Faculty for Biology and Biotechnology, Ruhr-University Bochum, Bochum, Germany

^5^Department of General Zoology and Neurobiology, Ruhr-University Bochum, Bochum Germany

***Corresponding author:**

*Martin Hadamitzky, PhD*

Institute of Medical Psychology and Behavioral Immunobiology

Center for Translational Neuro- Behavioral Sciences

University Hospital Essen, 45147 Essen, Germany

E-Mail: martin.hadamitzky@uk-essen.de

**
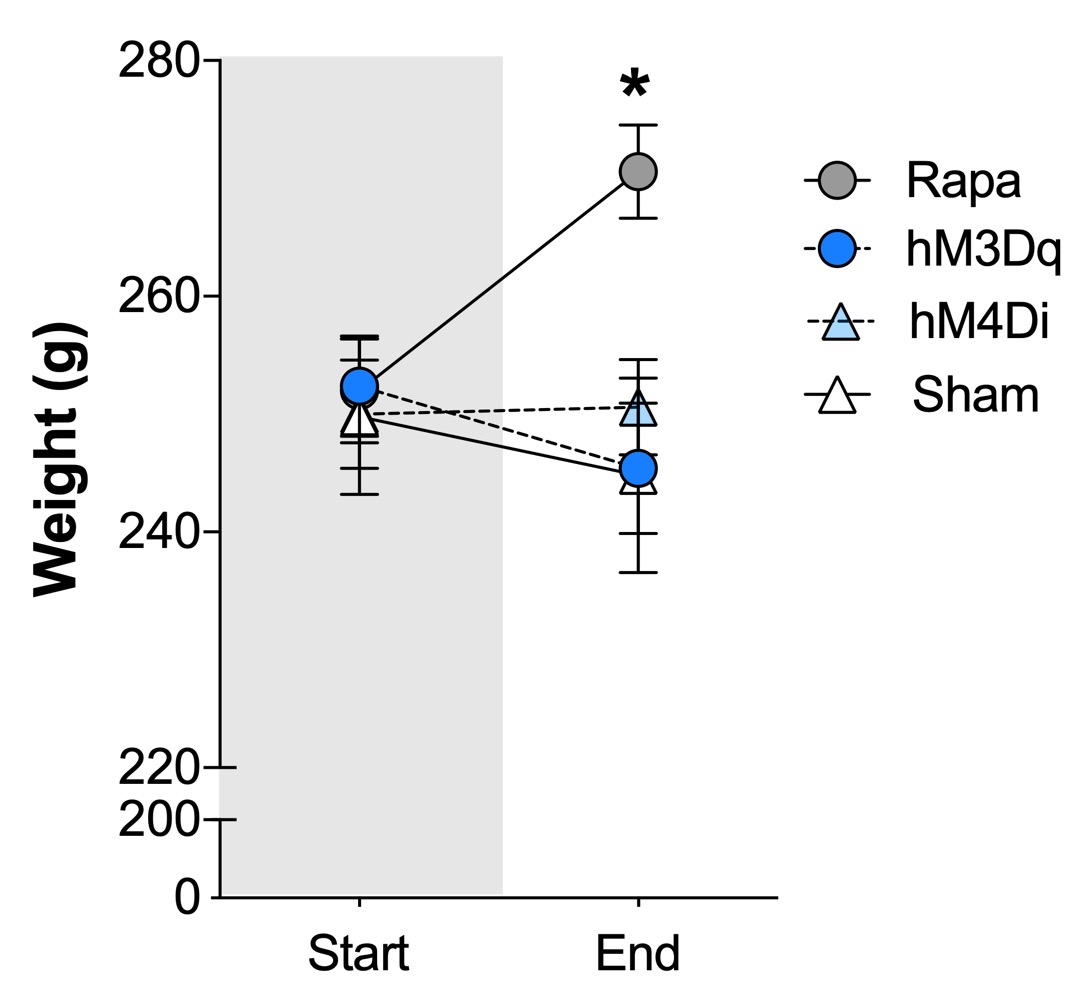
**

**Supplementary Fig. 1** Body weight development. All groups were administered with rapamycin (5 mg/kg) over the seven days except for the pharmacological control group (*Rapa*) which received vehicle instead. While the *Rapa* group displayed a significant body weight gain over that time period, the experimental groups (*hM3Dq, hM4Di, Sham*) showed stable body weight when compared to the initial weight before drug treatment (Start = before the first injection, End = after the last injection; ANOVA; Tukey’s test *p < 0.05, vs. *hM3Dq, hM4Di, Sham*). Data are means ± SEM (n=8-10/group).

**
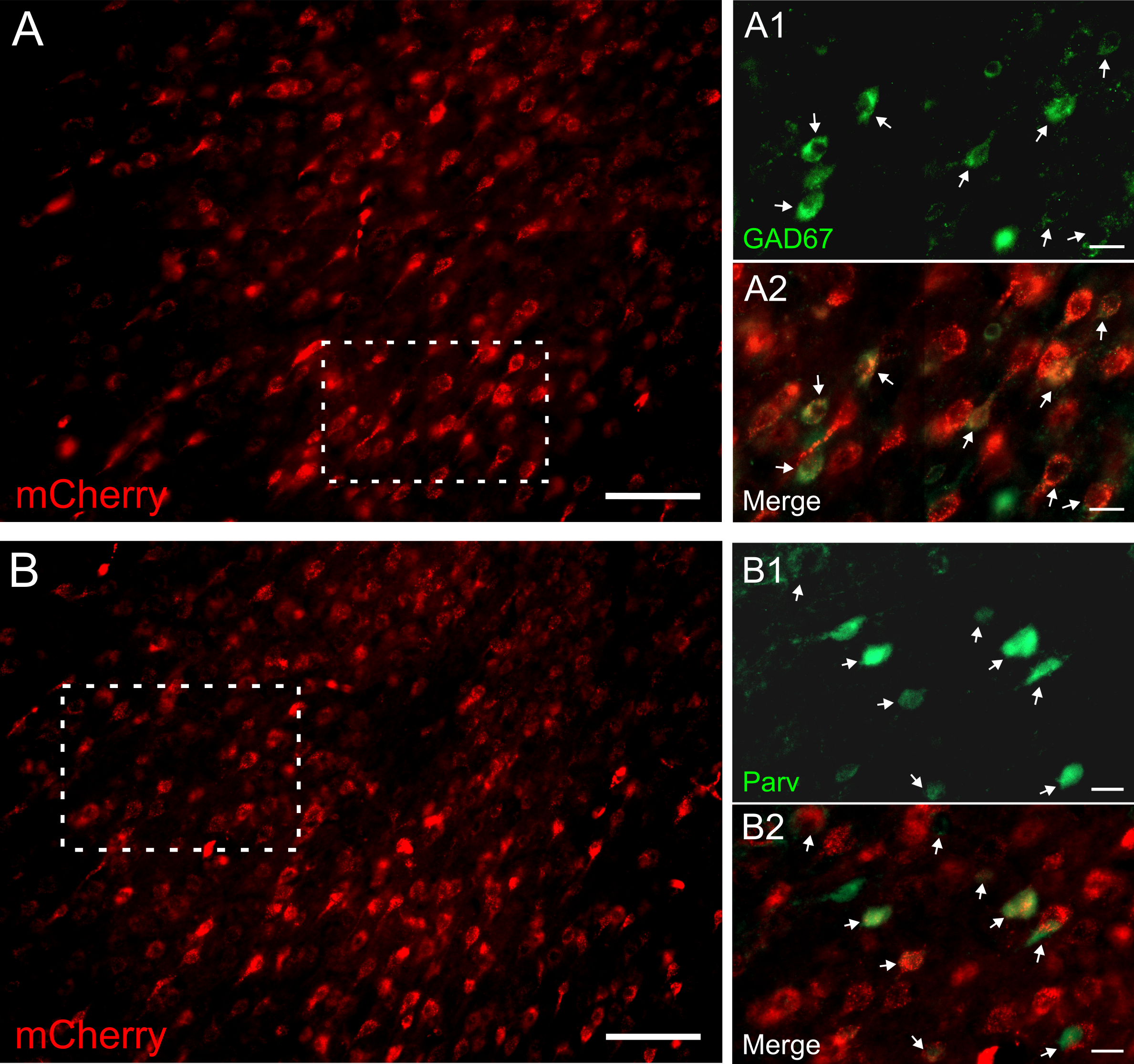
**

**Supplementary Fig. 2.** Immunofluorescent labeling of pAAV-CaMKIIa-hM4D(Gi)-mCherry with GAD67^+^ and Parv^+^ neurons in the insular cortex. (**A**) Expression of AAV-CaMKIIa-hM4D(Gi)-mCherry (red) in cortical neurons of the INC. Magnification: 20 x; Scale bar: 100 μm. (**A1, 2**) Magnified images of the dashed part showing colocalization of mCherry (red) and GAD67 (green) in the INC. Original magnification: 20 x. Scale bar: 20 μm. (**B**) Expression of AAV-CaMKIIa-*hM4D*(Gi)-mCherry (red) in cortical neurons of the INC. Magnification: 20 x. Scale bar: 100 μm. (**B1, 2**): Magnified images of the dashed part showing colocalization of mCherry (red) and Parv (green) in the INC. Arrows point out neurons co-labeled for the respective marker and AAV-CaMKIIa-*hM4D*(Gi)-mCherry (INC=insula cortex; GAD67=glutamate decarboxylase 67; Parv= parvalbumin).


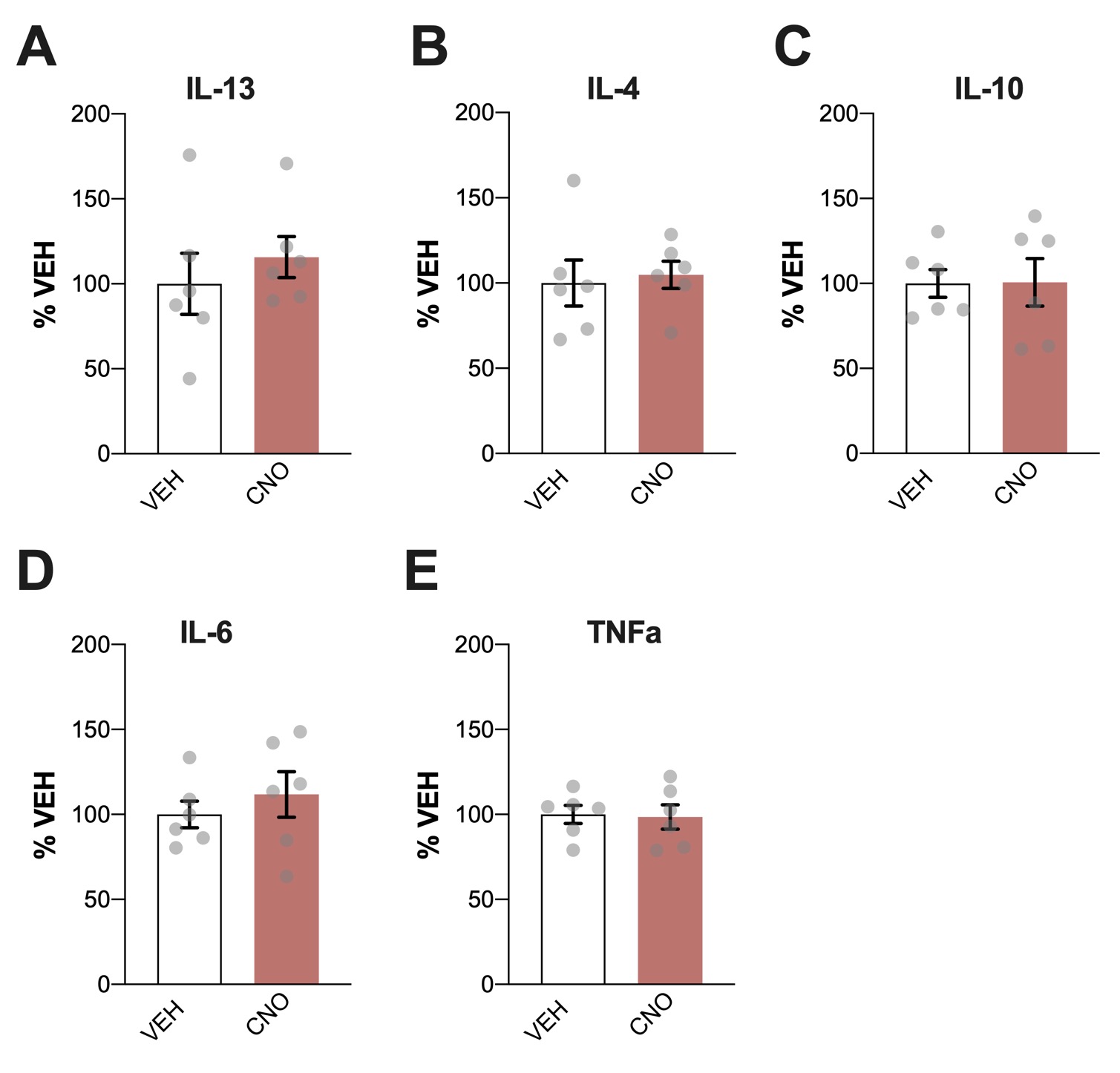


**Supplementary Fig. 3** Impact of CNO on cytokine production. One group received a single administration of CNO (1 mg/kg) and cytokine production of ex vivo stimulated splenocytes was compared with vehicle (VEH)-treated animals 90 min following injection. Single CNO treatment had no effects on peripheral immune responses. Data are means ± SEM (n=6/group; Unpaired t-test).
